# The relationship between uncertainty and acute procedure anxiety among surgical patients in Chinese mainland: the mediating role of resilience

**DOI:** 10.1186/s12888-023-05315-5

**Published:** 2023-11-01

**Authors:** Min Wu, Suwan Dai, Rong Wang, Silan Yang

**Affiliations:** 1 The First Hospital of Jiaxing, Affiliated Hospital of Jiaxing University, Jiaxing, Zhejiang China; 2 The graduate school of Zhejiang Chinese Medicine University, Hangzhou, Zhejiang China

**Keywords:** Anxiety, Resilience, Uncertainty, Mediation effect, Surgical patients

## Abstract

**Background:**

Surgery, as one of the main diagnostic and treatment methods, is a routine work in medical settings. Patients undergoing surgery often experience acute procedure anxiety due to uncertainty. There is ample evidence showing that uncertainty is a risk factor for the acute procedure anxiety in surgical patients. However, little is known about the psychological processes mediating this relationship. Therefore, this study aims to evaluate resilience as a mediator of the association between uncertainty and anxiety.

**Methods:**

A population-based cross-sectional survey with a convenience sampling method was conducted, involving 243 surgical patients in Jiaxing, Zhejiang province of China was carried out. Relevant data were collected by self-reporting questionnaires, including demographic characteristics questionnaire, Amsterdam Preoperative Anxiety and Information Scale (APAIS-C), Mishel’s Illness Uncertainty Scale (MUIS), Connor-Davidson Resilience Scale (CD-RISC). Pearson correlation analysis was employed to examine correlations between various variables. A path model was used to assess the mediation effect of resilience with respect to uncertainty and acute procedure anxiety.

**Results:**

In the path model, uncertainty have an indirect effect on acute procedure anxiety through resilience. The results suggest that resilience has a mediating role in uncertainty and acute procedure anxiety among surgical patients.

**Conclusions:**

These findings call for the development of interventions targeting the role of resilience in effectively predicting and preventing acute procedure anxiety and uncertainty among surgical patients.

## Introduction

On May 20, 2022, the General Office of the State Council of China have issued the National Health Plan for the 14th Five Year Plan [1]. In this plan, Clearly propose to improve the comprehensive and full cycle health service system, promoting mental health was highlighted. The mental condition can be changeable in different period and surroundings, especially for some special situation like before the surgery in hospital. According to the 2022 China Health Statistics Yearbook, the total number of surgeries nationwide in 2021 exceeds 75 million [2].

Surgery, as the most frequently used medical interventions, which also happens to be the critical cause of patient acute procedure anxiety. Acute procedure anxiety is an excessive fear of dental, medical, or surgical procedures that results in acute distress or interference with completing necessary procedures [3]. Previous studies have shown that acute procedure anxiety tends to peak before surgery during most procedures, with a prevalence of approximately 11–80% [3–5]. The acute procedure anxiety can cause many adverse reactions, such as the postoperative delirium, pain and emotional distress [6, 7], hypertension [8], even leading to the cancellation of the surgery. What is more, acute procedure anxiety is the most common predictor for more severe pain postoperatively [5, 9]and can worsen patients’ cognitive function, well-being, and quality of life [10]. During hospitalization, severe anxiety can reduce the sense of experience and even create a sense of fear for patients undergoing future anesthesia and surgeries. Because of the huge surgical volume, the number of anxious surgical patients has reached an undeniable level and can’t be ignored. As we know, the risk factors for acute procedure anxiety include invasiveness [11], treatment procedures [12], and also include uncertainty [3, 5, 13].

Uncertainty refers to a lack of the ability to determine things related to a disease [14]. It is a cognitive state that comes with symptoms, diagnosis, treatment, and prognosis related to the disease. The conceptual model of uncertainty is derived from and applied to nursing practice [14]. As an important link in the treatment of diseases, surgery is closely related to uncertainty because of lack of accurate information, complexity of the treatments, and loss of somatic functions due to surgery. There was a previous study [15]evaluated illness uncertainty, anxiety and depression among 363 Ophthalmic surgery patients from Zhongshan Ophthalmic Center in China and showed anxiety was positively significantly associated with illness uncertainty. In addition, researches show that uncertainty about the effectiveness of currently available treatments can trigger acute procedure anxiety [16]. Uncertainties and unfamiliarity stemming from surgery can exacerbate patients’ acute procedure anxiety [17, 18]. All of them can be seen that uncertainty is an important influencing factor in anxiety, and we should find the mechanism of uncertainty and provide theoretical basis for future interventions.

**Fig. 1:**
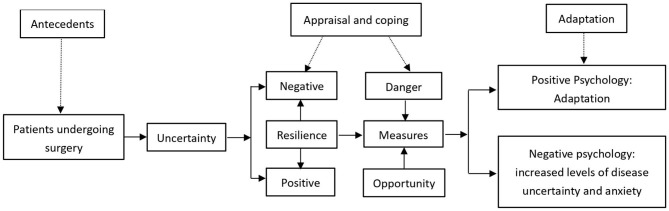
Liu’s illness uncertainty theory frame

The theory from Mishel’s uncertainty in illness consists of four major components [19]: (a) antecedents generating uncertainty, (b) appraisal of uncertainty, (c) coping with uncertainty, and (d) adaptation. Based on Mishel’s theory, Liu Dan [20] believed that as a stressor, surgery was also an antecedent that could cause patients to have uncertainty, while psychological resilience could affect patients’ responses during the appraisal and coping stages. Here is Liu’s theory frame in Fig. 1. Furthermore, most scholars believe that uncertainty of illness was the main factor that caused psychological pressure in patients and seriously affected their psychological resilience [14, 21].

Historically, the term ‘resilience’ has been used to conceptualize it as an individual’s relatively stable personality trait [22]. However, Connor and Davidson said resilience is actually a multidimensional characteristic that varies with age, time, environment, gender, and cultural origin, as well as individual living surroundings. It can be changed and improved with treatment [23]. It is considered to be a defense mechanism that can rapid recover from challenging events, then make people stronger from the stressful events [24]. In previous studies, resilience could played a moderating and mediating role in the association between coping styles and uncertainty [25], and another study [26] reported that the mediating and moderating roles of it in the relationship between anxiety, depression, and post-traumatic growth among breast cancer patients, which were conducive to providing new ideas and programs for improvement for these research populations. In addition, many researchers found that individuals with higher resilience tend to exhibit lower anxiety [27, 28], that means resilience and anxiety were negatively correlated. Based on the findings, it can be inferred that surgical patients with a higher level of resilience are more adept at recognizing the specific stressors they encounter and effectively utilizing suitable coping mechanisms. In summary, among surgical patients, resilience appears to act as a mediator in the relationship between uncertainty and anxiety. We expected a significant direct and indirect effect in our model.

Despite the growing evidence in other fields, much less is known about the relations between resilience and acute procedure anxiety in surgical patients with a sense of uncertainty. Based on previous research, we conducted a cross-sectional survey to investigate the correlation between resilience, uncertainty, and acute procedure anxiety in surgical patients, as well as the mediating effect of resilience. We aimed to provide a conceptual reference framework for prevention and intervention to help surgical patients cope with acute procedure anxiety.

Building and enlarging on previous results, we propose a model of the uncertainty on acute procedure anxiety that includes resilience as potential mediators based on Jiang Qianjin’s stress system model [29]. What the stress system model suggests is that there are many intermediary variables between stressors (surgery, illness certainty) and stress reactions (psychological, behavioral and physiological outcome), including social support, cognitive evaluation, personality and other psychological factors [29]. Following this model, this research hypothesize that the relationship between acute procedure anxiety(behavioral outcome) and uncertainty(stressor) is partially mediated by resilience(psychological factors), after considering context variables (e.g., demographics).

**Fig. 2:**
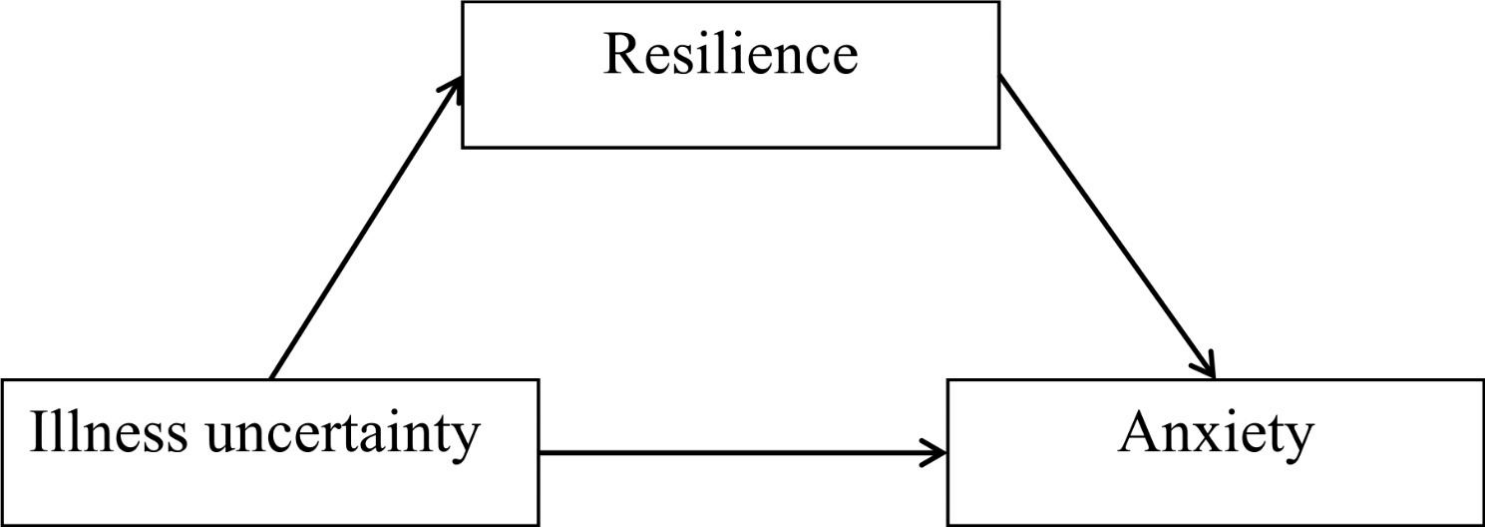
Theoretical model framework

There are following hypotheses we propose: (1) uncertainty is negatively related to resilience, while uncertainty is positively related to acute procedure anxiety; (2) resilience is negatively related to acute procedure anxiety; and (3) resilience mediates the relationship between uncertainty and acute procedure acute procedure anxiety. The theoretical hypothesis model as shown in Fig. 2. We hope to reveal the inner link of uncertainty, resilience and acute procedure anxiety, and look for new approaches to alleviate acute procedure anxiety related to uncertainty in the future.

## Methods

### Study population

A cross-sectional study with a convenience sampling method was conducted. Our study sample was made up of surgical patients in the pre-anesthesia room of a grade A general hospital in Jiaxing, a city located in Zhejiang province, southeastern China from November 7 to December 16, 2022. This study was approved by the Institutional Review Board at the local hospital (Number: 2022-KY-239).

### Procedure

The pre-anesthesia room is a temporary rest room waiting for surgery, where surgical patients are separated from their care givers and receive treatment before the procedure like artery puncture, and they usually stay there for about half an hour. Our survey was conducted in the pre-anesthesia room. Before filling out the questionnaires, patiently explained the purpose, significance, method, etc. of the questionnaires and told them entire survey will take 15–20 min to obtain the patient’s informed consent and signature. Young patients would like to use electronic devices such as mobile phones or iPads for online questionnaires(the web site: www.wjx.cn, an online survey platform popular in China ), while those who are not accustomed to screen, like the aged used paper questionnaires. For patients with reading disabilities, trained researchers conduct inquiries. Before the surgical patients completed the questionnaires, they were required to honestly and completely answer the questions on the questionnaires. All of them were told that the questionnaires results were only for scientifical research.

### Participants

Criteria for selecting the participants were as follows:(a) patients undergoing surgery; (b) Age greater than or equal to 18 years old; (c) they have clear consciousness and can express themselves correctly; and (d) they volunteered for the study. Correspondingly, the exclusion criteria were as follows: (a) patients undergoing emergency surgery; (b)Suffering from severe mental illness or cognitive impairment; (c) Communication barrier, unable to communicate normally with researchers or unable to complete the questionnaire.

The sample size was calculated according to formula for calculating sample size in cross-sectional surveys [30]. Considering a 20% loss of access rate and sampling error, the sample size was expanded to 230. We initially recruited 287 patients for the study. However, after excluding missing items and unqualified questionnaires(e.g. questionnaire filling time is less than 2 min, blank questionnaire), we excluded 44 questionnaires. Ultimately 243 were included in the analysis.

### Measures

The questionnaires used for this survey consisted of four sections (A: patient characteristics, B: preoperative anxiety, C: uncertainty, D: resilience).

#### Demographic characteristics questionnaire

A general information form included general sociodemographic information of patients and items that may be related to anxiety, such as age, gender, education level, occupation, tumor-related diagnosis.

#### Amsterdam preoperative anxiety and information scale (APAIS-C)

The Amsterdam Preoperative Anxiety and Information Scale (APAIS), which contains six items assessed the magnitude of patient’s preoperative anxiety which is the sum of anesthesia and surgery (APAIS-A-T, four items, score 4–20), and need for information (APAIS-I-T, two items, score 2–10), developed by Dutch Moerman in 1995 [31], Chinese version of the validated Amsterdam Preoperative Anxiety and Information Scale (APAIS-C) [32] was made by Le in 2019. Previously, the specifics and effectiveness of APAIS have been shown and validated in various languages and countries [33–36]. Le’s version resulted of this survey demonstrated that the reliability (Cronbach’s α) of the four anxiety items (“anxiety scale”) and of the two information items (“information scale”) were 0.898 and 0.806, respectively [32]. Based on the strong evidence of its reliability and validity, the APAIS was regarded as the optimal measure of preoperative anxiety. In this study, both the anxiety scale (four items) and the information scale (two items) showed high reliability (Cronbach’s α) of 0.947 and 0.946, respectively.

#### Mishel’s illness uncertainty scale (MUIS)

The Chinese version of the Illness Uncertainty Scale was translated and revised by Professor Xu [37] from Taiwan based on the Mishel’s Illness Uncertainty Scale (MUIS) [38], which is a self-rating scale. The scale uses a Likert 5-point rating, 1–5 indicating from strongly disagree to strongly agree, among which items 6, 7, 9, 23, and 25 are reverse scoring items; The scale score ranges from 25 to 125 points, and the high and low scores represent the high and low of uncertainty. The scale score is classified into three categories: low (25-58.3), medium (58.4–91.7), and high (91.8–125) uncertainty. The scale has two dimensions: uncertainty with 15 items and complexity with 10 items. The content validity index of this scale is 0.92, the internal consistency reliability Cronbach’s α value is 0.87. In this research, the Cronbach’s α value is 0.912.

#### Connor-davidson resilience scale (CD-RISC)

In 2003, CD-RISC was compiled by psychiatrists and behavioral scientists Dr. Connor and Davidson [23], then revised into Chinese [39]. The Chinese version consisted of 25 items, which are composed of three dimensions: resilience (items 11–23), strength (items 15, 78, 9, 10, 24, 25), and optimism (items 2, 3, 4, 6). It’s used to assess the level of psychological resilience of individuals and has been widely used. The scale scoring uses Likert’s 5-level scoring method. The numbers 0–4 correspond to never, rarely, sometimes, often and almost always respectively. The scale’s total score ranges from 0 to 100 points. According to the scale, the higher the score, the better the one’s psychological resilience. The Cronbach’s α coefficient of the translated Chinese version scale was 0.91.In this study, the Cronbach’s α of CD-RISC is 0.882.

### Statistical analysis

Statistical analysis was performed using SPSS 25.0 software. First of all, 44 missing data points were excluded after we checked for missing values, outliers and normality before data analysis. Secondly, starting to calculate the total score of each scale, APAIS-C, MUIS and CD-RISC exhibited an approximately normally distribution. T tests and ANOVA were used to determine the associations between patients characteristics and acute procedure anxiety. We examined relationships between variables by Pearson correlation analysis [40]. Third, the PROCESS macro for SPSS was used to analyze the hypothesized mediation model, the approach based on ordinary least-squares regression and the bootstrap method [41]. Bootstrapping does not require the assumption of normality of the sampling distribution, and it has higher power while maintaining reasonable control over the Type I error rate [42]. Non-standardized beta coefficients are calculated to reduce Type 1 errors due to distribution. Hayes’ SPSS macro program Process Model 4 was utilized to analyze the mediation effect among anxiety, uncertainty and resilience. We used 5,000 bootstrap resamples to calculate the 95% CI. If the interval did not include zero, the effect was statistically significant at p < 0.05.

## Results

### Characteristics of participants and differences in anxiety, uncertainty and resilience

The patients’ ages ranged from 21 to 85 years old (mean = 48.78, SD = 13.98), as shown in Table 1. Among them, 103 (42.4%) were male, 140 (57.6%) were female. the majority (64.6%) had full-time job. Further statistical tests revealed that surgical patients of different gender(t=-3.674 P < 0.001)and tumor-related diagnosis (t=-2.211 P = 0.028) had significant differences in anxiety. The results, as shown in Table 1, indicate that female patients showed higher levels of anxiety and uncertainty with lower level of resilience. The higher education level, the higher the scores for resilience. Patients with tumor-related diagnosis indicated higher level of anxiety and also uncertainty.

**Table 1:** Patients characteristics and differences in participants’ anxiety, illness uncertainty and resilience[M(SD)]

Variable	N(%)	Anxiety	t/F	P	Illness Uncertainty	t/F	P	Resilience	t/F	P
**Gender**										
Male	103(42.4)	7.73(4.22)	t=-3.674	0.000**	50.07(9.92)	t=-2.421	0.016*	67.20(8.79)	T = 2.768	0.006**
Female	140(57.6)	9.78(4.36)			53.59(12.08)			63.86(9.69)		
**Education level**										
Below primary school	16(6.6)	10.31(5.13)	F = 0.614	0.751	54.31(13.07)	F = 0.613	0.654	62.06(9.51)	F = 3.000	0.019*
Primary school	64(26.3)	9.16(4.94)			53.48(10.64)			63.28(10.48)		
Junior school	61(25.1)	8.49(4.61)			51.00(10.74)			65.02(8.27)		
High school	33(13.6)	8.88(3.60)			51.09(10.82)			64.97(7.42)		
College	69(28.4)	8.74(3.89)			51.75(12.36)			68.25(9.69)		
**Age**										
< 44	91(37.4)	9.27(3.93)	F = 0.450	0.739	51.54(11.58)	F = 0.582	0.627	66.66(9.99)	F = 1.465	0.225
45–59	97(39.9)	8.71(4.51)			52.16(10.81)			63.88(9.89)		
60–74	49(20.2)	8.53(4.93)			47.33(10.09)			65.22(7.47)		
> 75	6(2.5)	9.67(5.72)			52.10(11.33)			67.33(5.35)		
**Occupation**										
Unemployed	28(11.5)	10.00(4.42)	F = 0.994	0.372	52.29(10.87)	F = 1.105	0.333	63.93(9.98)	F = 0.321	0.726
Full-time	157(64.6)	8.81(4.24)			52.77(10.36)			65.44(9.68)		
Retiree	58(23.9)	8.66(4.83)			50.19(13.79)			65.48(8.59)		
**Tumor-related diagnosis**										
Yes	96(39.5)	8.41(4.37)	t=-2.211	0.028*	50.93(10.98)	t=-1.999	0.047*	66.17(9.66)	t = 1.836	0.068
No	147(60.5)	9.68(4.37)			53.89(11.67)			63.91(8.97)		

*<0.05, **<0.01

### Correlation analysis of anxiety, uncertainty and resilience

The basic descriptive data for anxiety, uncertainty and resilience are shown in Table 2. Surgical patients reported that mean (SD) total acute procedure anxiety (APAIS-A-T, range = 4–20) was 8.91 (4.41). High anxiety (APAIS-A-T > 10) was reported by 35.8% of subjects. MUIS(range = 25–125) and CD-RISC(range = 0-100) with a mean (SD) score of 52.10 (11.33) and 65.28(9.44) respectively. Pearson’s correlations showed that uncertainty was significantly negatively correlated with resilience (r = − 0.514, p < 0.001), while was significantly positively correlated with acute procedure anxiety (r = 0.382, p < 0.001).

**Table 2:** Descriptive statistics and correlation of anxiety, illness uncertainty and resilience (N = 243)

	1	2	*2.1*	*2.2*	3	*3.1*	*3.2*	*3.3*
1 Anxiety								
2. Illness uncertainty	0.40_**_							
*2.1 Uncertainty*	0.40_**_	0.96_**_						
*2.2 Complexity*	0.25_**_	0.77_**_	0.60_**_					
3. Psychological resilience	-0.36_**_	-0.43_**_	-0.41_**_	-0.35_**_				
*3.1 Resilience*	-0.34_**_	-0.37_**_	-0.35_**_	-0.28_**_	0.94_**_			
*3.2 Optimism*	-0.25_**_	-0.33_**_	-0.31_**_	-0.27_**_	0.67_**_	0.48_**_		
*3.3 Strength*	-0.32_**_	-0.41_**_	-0.39_**_	-0.35_**_	0.81_**_	0.66_**_	0.50_**_	
M	8.91	52.10	31.70	20.4	65.28	32.49	9.81	22.98
SD	4.41	11.33	8.47	3.81	9.44	5.60	1.99	3.05

N = 243. _*_P< 0.05, _**_P<0.01

### Mediating role of resilience on uncertainty and acute procedure anxiety

Table 3 demonstrates that all individual paths between the key variables in the model were found to be significant even after considering the covariates. According to the model, there is a positive correlation between uncertainty and anxiety (β = 0.1361, 95% CI: 0.0904 ~ 0.1818), while high resilience is negatively correlated with anxiety (β=-0.1016, 95% CI: -0.1637~-0.0395).The direct impact of uncertainty on anxiety was first investigated using regression analysis. The results showed a significant positive relationship between uncertainty and anxiety scores (β = 0.382, SE = 0.023, t = 6.420, p < 0.001).

**Table 3:** Results of the regression analyses testing the mediating effect of resilience in the relationship illness uncertainty and anxiety (N = 243)

Predictors	Beta	SE	t	P	95%CI	
					LLCI	ULCI
Consent	8.4495	3.1355	2.6948	0.0075**	2.2726	14.6264
Resilience	-0.1016	0.0315	-3.2251	0.0014**	-0.1637	-0.0395
Illness uncertainty	0.0941	0.0262	3.5930	0.0004**	0.0425	0.1457
Gender	1.2722	0.5335	2.3846	0.0179*	0.2212	2.3233
Tumor-related diagnosis	0.4610	0.5348	0.8620	0.3896	-0.5926	1.5145

Note: * P < 0.05; **P < 0.01, Unstandardized regression coefficients (Beta) with standard error (SE) in parentheses are presented, CI Confidence Interval

**Fig. 3:**
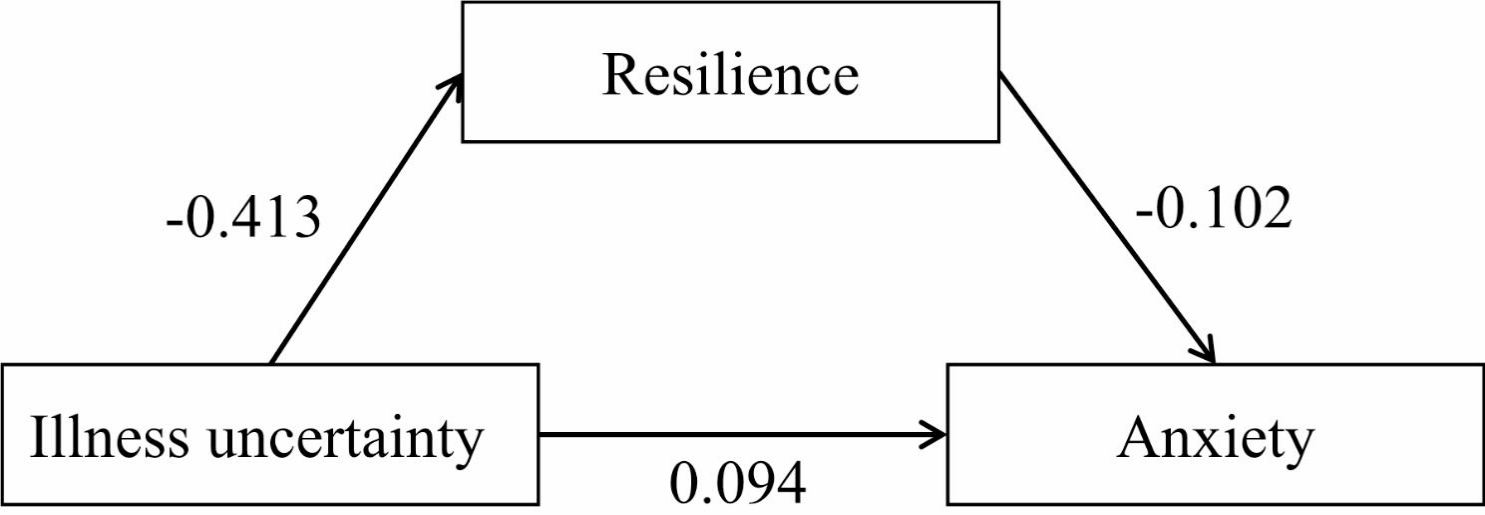
Resilience as the mediator between illness uncertainty and acute procedure anxiety

After controlling for covariates such as gender and tumor-related diagnosis, the mediating effect of resilience on the relationship between uncertainty and acute procedure anxiety was examined. The results of Model 4 mediation model showed that uncertainty had a significant positive effect on acute procedure anxiety (β = 0.094, SE = 0.062, t = 3.593, p < 0.001), uncertainty had a significant positive effect on resilience (β =- 0.413, SE = 0.047, t =-8.839, p < 0.001), and resilience had a significant negative effect on acute procedure anxiety (β = -0.102, SE = 0.032, t =-3.225, p < 0.01), as shown in Fig. 3.

Bootstrap resampling 5,000 times to test the model analysis results showed that in the “uncertainty →resilience → anxiety” path, the effect value of resilience was 0.04 [95%CI (0.02, 0.07)], and the 95%CI did not include 0, indicating that the mediating effect was established. Therefore, the hypothesis was confirmed. Uncertainty can not only directly affect anxiety but also indirectly affect anxiety through resilience. The total effect was 0.136, the direct effect was 0.094, and the mediating effect was 0.042. The mediating effect accounted for 30.88%. See Table 4.

**Table 4:** Bootstrapped point estimates with standard errors and 95% confidence intervals for all indirect effects between illness uncertainty and anxiety (N = 243)

Items	Beta/Effect	SE	t	P	95%CI	
					LLCI	ULCI
**Path way**						
Illness uncertainty◊Anxiety	0.1361	0.0232	5.8684	0.0000^**^	0.0904	0.1818
Illness uncertainty◊Resilience	-0.4128	0.0467	-8.8389	0.0000^**^	-0.5048	-0.3208
Resilience◊Anxiety	-0.1016	0.0315	-3.2251	0.0014^**^	-0.1637	-0.0395
**Effects**						
Direct effect	0.0941	0.0262	3.5930	0.0004^**^	0.0425	0.1457
Indirect effect	0.0419	0.0120			0.0198	0.0675
Total effect	0.1361	0.0232	5.8684	0.0000^**^	0.0904	0.1818

Bootstrap resample = 5,000; Control variables (gender and tumor-related diagnosis) are included in the analysis. If the CI does not include zero, the effect is statistically significant at p < 0.05.

*Abbreviations*: SE Standard error, LLCI Lower Level of Confidence Interval, ULCI Upper Level of Confidence Interval.

## Discussion

Due to the high incidence of acute procedure anxiety, understanding psychological mediators of the association between uncertainty and acute procedure anxiety appears essential to identify appropriate targets for novel treatment approaches that can be offered to surgical patients with anxiety. In this study we sought to examine the contribution of resilience to anxiety in surgical patients. We predicted that (I) uncertainty would be positively associated with the incidence of acute procedure anxiety; (II) resilience is negatively related to acute procedure anxiety; (III) the association between uncertainty and acute procedure anxiety would be partially related to resilience.

### Descriptive analysis and social characteristics of surgical patients for acute procedure anxiety, uncertainty and resilience

Our study shows that the incidence of acute procedure anxiety was 35.8%, this is close to the research results of Le’s 36.6% [32]. The average total score of MUIS was 52.1 ± 11.3, similar to Dong’s 52.22 ± 12.51 [43].This finding demonstrates that in the pre-anesthesia room, patients undergoing surgery experienced significant anxiety when facing the unfamiliar environment and the uncertainty brought by the upcoming anesthesia and surgery. This finding is consistent with Choy’s [3] and Bailey’s previous research [44]. Consequently, the importance of psychological care for surgical patients in the pre-anesthesia room cannot be overlooked.

As the previous research [45, 46]discovered, this study found that in the incidence of acute procedure anxiety, female are more likely to be than male. In the literatures, there may be three reasons for this: first, women are usually more sensitive and emotive nature of their treatment [47]; second, males traditionally cannot easily express their weaknesses [48], and third, based on experimental studies [49], it has been suggested that fluctuations in estrogen and progesterone levels may contribute to the differences observed in female patients. Tumor-related diagnosis have also been identified as risk factors for anxiety previously. Gonzalez and colleagues indicated that anxiety are prevalent and occur more frequently in people who have been diagnosed with cancer compared with healthy controls [45]. It may be due to the dependence of tumor staging, diagnosis, and characterization on postoperative histopathological results, which can exist more uncertainty and lead to higher levels of anxiety. Tumor-related diagnosis was shown to be an impact factor for the development of anxiety in the univariate model for surgical patients (p = 0.028) in our study. However, the correlation was not confirmed in the multivariate model.

The average total score of CD-RISC was 65.28 ± 9.44, a little higher than Han’s result 64.89 ± 10.36 [25], but not as high as general population. There were some studies revealed that resilience can be viewed as a major mental defense mechanism and a promotable protective factor [50, 51], it can buffer the negative influence of adversity and be conductive to preventing individuals from high stress and psychological disturbance [52]. However, when individuals become ill and require anesthesia for surgery, the uncertainty surrounding both the anesthesia and the surgical procedure (such as potential accidents and risks during surgery, as well as side effects and adverse reactions to anesthesia) becomes a significant source of stress. This stressor can greatly impact patients’ psychological resilience, particularly those in the low-resilience category [53]. Therefore, interventions to improve resilience are necessary for patients in this group.

### Correlation between acute procedure anxiety, uncertainty and resilience

As predicted, uncertainty and acute procedure anxiety were substantially positively associated, in that higher levels of uncertainty corresponded with more severe anxiety. This is in line with recent studies results [45, 54]. In the current research, there was a negative correlation between anxiety and resilience. This means that the lower the resilience is, the higher the anxiety will be.

### Mediating role of resilience on uncertainty and acute procedure anxiety

Based on Jiang Qianjin’s stress system model, this study sought to describe the mediating role of resilience between uncertainty and acute procedure anxiety. In addition, the stimulus intensity of events (disease, surgery) to people is not only related to the events themselves, but also to the different emotional stability among different surgical patients [40]. Resilience plays a very important role in this process.

Our study noted that uncertainty not only have direct effects on acute procedure anxiety but also have indirect effects on acute procedure anxiety via resilience. Several reports have shown that resilience played a mediating and moderating role in the relationship between stress with depression and anxiety, negative life events with aggression [40, 55]. Resilience is a crucial factor to consider when examining the relationships between distress and anxiety [56]. Liu’s illness uncertainty model [20] proposes that an individual’s appraisal and coping measures mediate the outcomes of adaptation, and these measures are influenced by their level of resilience. In the context of anesthesia and surgery, which often generate uncertainty, resilience plays a key role in coping with stressors. Labrague [27] found that resilient individuals were more likely to report lower anxiety levels. Conversely, Mexican dialysis patients with lower psychological resilience experienced higher levels of anxiety [57]. Kumpfer’s resilience model [58] suggests that resilience is one of the most important factors for individuals in dealing with stressful events. This finding implies that improving resilience could be an effective approach to preventing acute procedure anxiety related to uncertainty in surgical patients.

As we know, resilient individuals not only adapt to difficulties, but also strive to improve by improving [59].In surgical patients, resilience was a crucial mediator of the relationship of uncertainty with anxiety, which might reveal a deeper understanding of the mechanism by which uncertainty affects anxiety and optimization of intervention measures based on the previous researches. To address this, we call for that not only the care givers, but also the policy makers should tailor interventions and managements to improve the surgical patients’ resilience as following: multicomponent interventions [60] (such as informational support, emotional support, appraisal support, and instrumental support), psychotherapy methods such as brief Cognitive Behavior Therapy(CBT) [61] (which is applied as first-line treatment in anxiety), a combination of effective preoperative education, involvement of social support [62], and individualized communication [18].

Ungar [63] suggests psychologists are now unequivocal that systemic influences play an equally important role as individual factors to positive outcomes. And also he says resilience is the methods to achieve functional outcomes such as long lasting mental health [63]that not be influenced by the operative procedure. Thus, we recommend that resilience could be an evaluation content [64] and also be an evaluating indicator to evaluate the effectiveness of intervention measures to acute procedure anxiety related uncertainty.

In summary, as health providers, we should provider not only the treatment and nursing but also adequate resources just like peer and medical staff support, which can be used one to one or with groups, building a warm and comfortable infrastructure to influence the resilience before the surgery. We suggest that resilience may be a significant risk factor for surgical patients with uncertainty. The findings of the present study offer an additional dimension of how patients are facing such excruciating challenges.

### Limitations and future research

We should keep in mind that our study may suffer from a selection bias because our patients are all from one hospital in Zhejiang province and cannot represent all surgical patients in China.

Secondly, this study only focused on the effect of psychological resilience on the relationship between uncertainty and anxiety, while psychological resilience did not fully mediate the relationship between uncertainty and acute procedure anxiety, mechanisms of other mediating variables can be explored in future research to provide more and better interventions.

Finally, we use this mediating understanding to distil pointers for mental health practices and future research agendas to alleviate anxiety related to uncertainty.

- According to the individual resilience, increase focus on promoting patient’s access to the resources that increase resilience.
- Tailor interventions that improve resilience to different patients.
- Pay attention to gender and tumor-related diagnosed differences in the factors that promote resilience and the impact of risk on developmental outcomes.
- Encourage multidisciplinary team members to work together to promote resilience to ensure multiple systems are influenced simultaneously.
- Encourage policy makers to consider the factors that promote resilience in addition to those that prevent pre-anxiety.

## Conclusion

Our research shows that the association of uncertainty and acute procedure anxiety can be explained when resilience as mediator among surgical patients. In addition, uncertainty negatively affected resilience and positively affected acute procedure anxiety, whereas resilience played a protective role between uncertainty and acute procedure anxiety. The study revealed preliminary relationship mechanisms for uncertainty, resilience and acute procedure anxiety in surgical patients. In future research, clinical interventions may include both the development of resilience-focused interventions aiming at promoting individual resilience for surgical patients, who have been affected by uncertainty.

## Data Availability

The datasets used and analysed during the current study are available from the corresponding author on reasonable request.

## Abbreviations

APAIS-C: Chinese version of Amsterdam Preoperative Anxiety and Information Scale
MUIS: Mishel’s Uncertainty Scale ;
CD-RISC: Connor-Davidson Resilience Scale;
CBT: Cognitive Behavior Therapy;
SE: Standard errors
CI: Confidence interval
